# Modifizierte Masquelet-Plastik: Technik der induzierten Membran im Wandel der Zeit

**DOI:** 10.1007/s00113-024-01474-6

**Published:** 2024-08-07

**Authors:** C. Fischer, S. Schipper, S. Langwald, F. Klauke, P. Kobbe, T. Mendel, M. Hückstädt

**Affiliations:** 1 Klinik für Unfall- und Wiederherstellungschirurgie, BG Klinikum Bergmannstrost Halle, Merseburger Straße 165, 06112 Halle (Saale), Deutschland; 2 Klinik für Unfall‑, Hand- und Wiederherstellungschirurgie, Universitätsklinikum Halle, Ernst-Grube-Straße 40, 06120 Halle (Saale), Deutschland

**Keywords:** Non-Union, Pseudarthrose, Diamond-Konzept, Induzierte Membran, Knochendefekt, Fractures, ununited, Pseudarthrosis, Diamond concept, Induced membrane, Bone defect

## Abstract

Die Rekonstruktion langstreckiger Knochendefekte infolge von primär traumatischen oder sekundär infektions- oder tumorbedingten Substanzverlusten stellt nach wie vor eine chirurgische Herausforderung dar. Die Kallusdistraktion über Segmenttransport, der vaskularisierte Knochentransfer und die induzierte Membrantechnik (IMT) stellen etablierte Verfahren der Rekonstruktion dar. In den letzten Jahrzehnten erfreut sich die IMT aufgrund ihrer Praktikabilität, Reproduzierbarkeit und Zuverlässigkeit zunehmender Popularität. Gleichsam erfuhr die Originaltechnik eine Vielzahl von Modifikationen. Die Ergebnisse stellen sich als entsprechend heterogen dar. Diese Übersicht soll die wesentlichen Grundprinzipien der IMT darlegen und einen Überblick über die verschiedenen Modifikationen und ihre Komplikationen geben.

## Abstract (en)

The reconstruction of long bone defects as a result of primary traumatic, secondary infection or tumor-related loss of substance continues to represent a surgical challenge. Callus distraction via segment transport, vascularized bone transfer and the induced membrane technique (IMT) are established methods of reconstruction. In recent decades IMT has experienced increasing popularity due to its practicability, reproducibility and reliability. At the same time, the original technique has undergone numerous modifications. The results are correspondingly heterogeneous. This overview is intended to explain the basic principles of IMT and to provide an overview of the various modifications and their complications.

## Lernziele

Nach der Lektüre dieses Beitrags

- können Sie die Grundprinzipien der Masquelet-Technik benennen.
- sind Sie in der Lage, die Bedeutung des Verfahrens im Rahmen der Rekonstruktion knöcherner Defekte verschiedener Ätiologie einzuordnen.
- können Sie verschiedene Modifikationen der Masquelet-Technik, verglichen zur originalen Technik, benennen.
- fühlen Sie sich sicher darin, die möglichen Ursachen für ein Versagen des Verfahrens zu identifizieren.
- können Sie die beschriebenen Modifikationen im eigenen Patientenklientel anwenden.

## Historie

Die Technik der induzierten Membran (IMT) wurde erstmals 2000 von Masquelet et al. anhand einer Fallserie von 35 Patienten beschrieben [1]. Es handelt sich um ein **zweizeitiges Verfahren**

zweizeitiges Verfahren

zur Behandlung großer metadiaphysärer **Knochendefekte**

Knochendefekte.

### Erste Phase

In der ersten Phase erfolgen ein **Weichteil-Débridement**

Weichteil-Débridement

sowie die **Resektion**

Resektion

von minderperfundierten bzw. nekrotischen Knochenarealen. Die resultierende knöcherne Defektzone wird unter Verwendung eines **Zement-Spacers**

Zement-Spacers

aus Polymethylmethacrylat (PMMA) ausgefüllt. Die Stabilisierung wird durch eine überbrückende Montage eines Fixateur externe gewährleistet. In einem Zeitfenster von ca. 8 Wochen bildet sich um den Spacer eine gut **vaskularisierte Pseudosynovialmembran**

vaskularisierte Pseudosynovialmembran

aus.

### Zweite Phase

In der zweiten Phase der Defektauffüllung erfolgen im Rahmen der Revision zunächst die vorsichtige Inzision der Membran und die Entfernung des Platzhalters. Der knöcherne Defekt wird anschließend mit autologer, morcellierter **Beckenkammspongiosa**

Beckenkammspongiosa

aufgefüllt und mithilfe der internen oder externen **Fixation**

Fixation

stabilisiert [1, 2, 3]. Masquelet et al. gelang es, mithilfe dieser Technik in einer Serie von 35 beschriebenen Fällen Knochendefekte einer Länge von 5–24 cm zu rekonstruieren. Laut Autoren konnte eine **hilfsmittelfreie Vollbelastung**

hilfsmittelfreie Vollbelastung

im Mittel nach achteinhalb Monaten erreicht werden [1]. Die erfolgreiche Anwendung der IMT im Rahmen posttraumatischer Knochendefekte, septischer bzw. aseptischer Pseudarthrosen, Tumorresektionen oder der Behandlung der Osteomyelitis wurde bereits in mehreren Studien dokumentiert [4, 5, 6, 7].

## Allgemeine Aspekte der Knochenheilung

Die Knochenheilung basiert auf dem komplexen Zusammenspiel einer Vielzahl von Faktoren auf molekularer Ebene in Verbindung mit physiologischen und biomechanischen Prinzipien. Im Allgemeinen sind **Signalmoleküle**

Signalmoleküle,

**Osteoprogenitorzellen**

Osteoprogenitorzellen

und die extrazelluläre Matrix von wesentlicher Bedeutung. Die Signalmoleküle lassen sich im Wesentlichen vier Gruppen zuordnen: proinflammatorischen Zytokine, Wachstumsfaktoren (z. B. Transforming Growth Factor β [TGF-β], „bone morphogenetic protein“ [BMP]), Metalloproteinasen und angiogenen Faktoren. Diese biologisch aktiven Moleküle können durch die verschiedenen im Frakturhämatom vorkommenden Zelltypen (Endothelzellen, Thrombozyten, Makrophagen, Monozyten, mesenchymale Stammzellen) sezerniert werden und ihre osteoinduktive Wirkung auf die Osteoprogenitorzellen entfalten. Das Frakturhämatom und eine suffiziente Vaskularisation sind essenzieller Bestandteil des knöchernen Reparaturprozesses. Die **extrazelluläre Matrix**

extrazelluläre Matrix

bildet das Grundgerüst für diese molekularen Interaktionen. Im klinischen Kontext werden heutzutage verschiedene osteokonduktive Strukturen, angereichert mit osteogenen bzw. osteoinduktiven Faktoren, verwendet, um die Knochenheilung zu fördern. Dazu zählen u. a. allogene bzw. xenogene Knochentransplantate, demineralisierte Knochenmatrix, Kollagen, Hydroxylapatit, bioaktive Gläser und Keramik auf Kalziumbasis [8].

## Diamond-Konzept

Um die komplexen molekularen Mechanismen im klinisch-operativen Kontext adäquat umzusetzen, bedarf es eines praktikablen Konzepts. Diesem Umstand wurde in dem von Giannoudis et al. 2007 publizierten Diamond-Konzept zur Behandlung von Knochendefekten und **Pseudarthrosen**

Pseudarthrosen

Rechnung getragen [9]. Es basiert auf 5 Kriterien zur Optimierung der biologischen und biomechanischen Prozesse der **Knochenheilung**

**Abb. 1:**
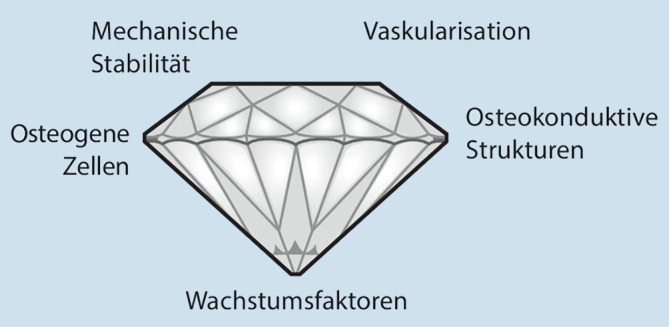
Diamond-Konzept. (Aus Miska und Schmidmaier [5])

Knochenheilung:

osteogene Zellen, osteoinduktive Strukturen, Wachstumsfaktoren, Vaskularisation und mechanische Stabilität ([10]; Abb. 1).

Die **mechanische Stabilität**

mechanische Stabilität

spielt eine entscheidende, häufig vernachlässigte bzw. mitunter nichtverstandene Rolle in der Behandlung knöcherner Defekte und Pseudarthrosen. Anhand verschiedener Untersuchungen konnte gezeigt werden, dass die Knochenbruchheilung zwar eine ausreichende Stabilität erfordert, für eine Sekundärheilung gleichermaßen aber auch **Mikrobewegungen**

Mikrobewegungen

im Frakturspalt gewährleisten muss, um die Stimulation und Differenzierung von Fibro‑/Chondro- und Osteoblasten anzuregen [11]. Die **Konsolidierungszeit**

Konsolidierungszeit

von Knochendefekten bzw. Pseudarthrosen stellt sich deutlich länger dar, verglichen mit der primären physiologischen Knochenheilung. Dies gilt es bei der Wahl des Osteosyntheseverfahrens (absolute vs. relative Stabilität) unter der Gefahr eines mechanischen Versagens des Konstrukts zu beachten.

## Schlüsselrolle der induzierten Membran

Der IM wird, neben allen anderen genannten Faktoren, eine Schlüsselrolle zuteil. Sie fungiert nicht nur als **physikalische Barriere**

physikalische Barriere

und verhindert die Resorption von Knochentransplantaten, sondern auch als **Bioreaktor**

Bioreaktor.

Sie fördert die Knochenheilung durch Revaskularisation („vascular endothelial growth factor“, VEGF), Sekretion von Wachstumsfaktoren (TGF‑β_1_; BMP-2) sowie durch Konzentration mesenchymaler Stammzellen (MSC) mit osteogener Potenz [12, 13].

### Merke

Die Prinzipien des Diamond-Konzeptes müssen bei der Behandlung von Pseudarthrosen umgesetzt werden.

## Modifikationen

Die ursprüngliche Technik der induzierten Membran wurde in den letzten Jahren mehrfach modifiziert. In der septisch-rekonstruktiven Chirurgie erfolgte zunehmend die Verwendung antibiotikabeschichteter oder imprägnierter Spacer. Die **Reamer-Irrigator-Aspirator-Technik**

Reamer-Irrigator-Aspirator-Technik

(RIA-Technik) wurde für die Gewinnung des spongiösen Autotransplantates aus dem Markraum langer Röhrenknochen entwickelt [14, 15, 16]. Zudem trat die interne Osteosynthese auch im Rahmen des Ersteingriffs zunehmend in den Vordergrund. **Knochenersatzmaterialien**

Knochenersatzmaterialien

aus Hydroxylapatit oder Trikalziumphosphat sowie demineralisierte Knochenmatrix („demineralized bone matrix“, DBM) bzw. demineralisierter Rinderknochen („demineralized bovine bone“, DBB) wurden dem Transplantat mit dem Ziel, das Füllvolumen zu vergrößern, zugesetzt. Knochentransplantate (allogen/xenogen), bioaktive Gläser und Keramik auf Kalziumbasis wurden als unterstützende Gerüststrukturen eingesetzt. Zur Optimierung der Osteoinduktivität erfolgte die Zugabe von Wachstumsfaktoren [14, 17, 18, 19, 20].

## Aktuelle Masquelet-Technik

In der aktuellen Literatur gibt es wenig Evidenz bezüglich der Konsolidierungsraten und des Patienten-Outcome [4, 14, 17]. Es finden sich einige retrospektive bzw. prospektive Fallserien zur Anwendung der IMT. Hinsichtlich der Methodik und der Erfolgsraten der Masquelet-Technik stellen sich die Ergebnisse dieser Studien allerdings ausgesprochen heterogen dar. Auch das empfohlene Zeitintervall zwischen den einzelnen Schritten variiert.

**Abb. 2:**
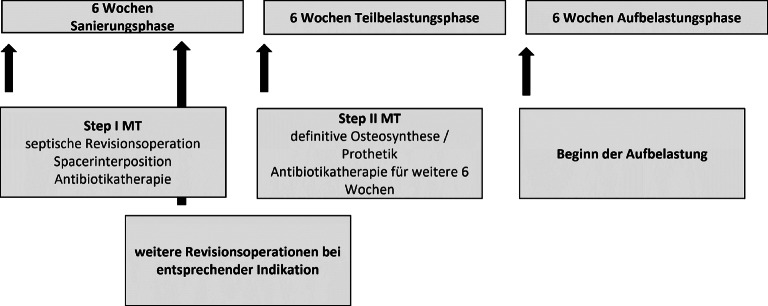
Ablauf der chirurgischen Phasen der Masquelet-Technik (*MT*) innerhalb der eigenen Klinik

Einige Autoren postulieren ein Intervall zwischen 4 und 8 Wochen, um eine adäquate Infekteradikation, Weichteilkonditionierung und Ausbildung einer robusten Membran zu ermöglichen [21]. Aus eigener Erfahrung der Autoren hat sich ein 6‑wöchiges Intervall zwischen den entsprechenden Phasen bewährt (Abb. 2).

Ein standardisierter Algorithmus zur Behandlung segmentaler Knochendefekte mithilfe der IMT existiert gegenwärtig nicht [18, 22, 23].

## Aktuelle Literatur

### Wirksamkeit

Die Arbeitsgruppen um Morreli et al. und Mi et al. publizierten 2016 bzw. 2022 die ersten umfangreichen „systematic reviews“ und Metaanalysen zur Wirksamkeit der IMT. Morelli et al. schlossen 17 Studien (*n* = 427 Patienten) in ihre Analyse ein. Die häufigsten Ursachen knöcherner Segmentdefekte waren posttraumatische Defekte (aseptische Pseudarthrosen, knöcherne Defekte), Infektionen (Osteomyelitis, Infektpseudarthrosen) und Tumoren. Nach Anwendung der Masquelet-Technik konnte eine vollständige knöcherne Konsolidierung der Segmentdefekte in 90 % der Fälle erzielt werden. **Revisionseingriffe**

Revisionseingriffe

wurden in 18 % der Fälle berichtet. Ursächlich waren eine **unzureichende Infekteradikation**

unzureichende Infekteradikation

und Pseudarthrosen. In 67,2 % der Fälle war die Tibia betroffen, die Fibula in 12,9 % der Fälle und das Femur in 19,4 % der Fälle. Die Größe der knöchernen Defekte betrug im Mittel 5,5 cm [14]. Das Intervall zwischen den beiden Phasen der IMT betrug durchschnittlich 44 Tage [14].

Mi et al. veröffentlichten vergleichbare Ergebnisse der IMT-Anwendung nach einer Analyse von insgesamten 41 Studien (*n* = 677 Patienten). Eine **vollständige Ausheilung**

vollständige Ausheilung

knöcherner Defekte oben genannter Entität konnte in 92 % der Fälle erreicht werden. Persistierende Infektionen und Pseudarthrosen stellten auch in dieser Metaanalyse die häufigsten Gründe eines komplikativen Verlaufes dar und führten in 22 % der Fälle zu einem Revisionseingriff. **Tibia**

Tibia

(59 %) und **Femur**

Femur

(23 %) waren die häufigsten Defektlokalisationen. Die durchschnittliche **Defektstrecke**

Defektstrecke

betrug 6,3 cm. Der Zeitraum zwischen den beiden Phasen der IMT betrug im Mittel 76 Tage [17].

### Antibiotikabeladene Spacer

Morelli et al. konstatierten, dass im Rahmen des Débridements (erste Phase) mehrheitlich antibiotikahaltige PMMA-Spacer verwendet wurden (62,5 %). Die Studienlage in Bezug auf deren Verwendung ist heterogen. Fung et al. untersuchten die Häufigkeit der Verwendung antibiotikahaltiger PMMA-Spacer nach Analyse von 48 Studien (*n* = 1373 Patienten).

Diese wurden in 69 % der Fälle verwendet. Zur Anwendung kamen hauptsächlich **Vancomycin**

Vancomycin

und **Gentamicin**

Gentamicin,

singulär (34 %) oder kombiniert (38 %) [24]. Sowohl der positive Effekt des beigefügten Antibiotikums auf die Infekteradikation als auch die zytotoxischen Eigenschaften einiger antiinfektiver Substanzen und deren Auswirkung auf die mesenchymalen Stammzellen sowie die Osteogenese sind weiterhin umstritten. Eine Empfehlung zum Einsatz antibiotikahaltiger PMMA-Spacer existiert gegenwärtig nicht [20, 23, 25]. Im eigenen Vorgehen hat sich die Verwendung von mittelviskosem PMMA mit Gentamicin bewährt.

### Fixationsmethoden

Anders verhält es sich mit der Fixationsmethode und der Entnahmestelle der Transplantate im Rahmen der zweiten Phase der IMT. Angewendet werden zumeist interne, externe oder kombinierte **Osteosyntheseverfahren**

Osteosyntheseverfahren

[14, 17]. Der Erfolg der IMT wird maßgeblich durch eine suffiziente mechanische Stabilität beeinflusst. Zudem bedarf es einer ausreichend **stabilen Füllstruktur**

stabilen Füllstruktur

des Transplantats in der Kavität der IM, um einem Sedimentationseffekt entgegenzuwirken [22]. Andrzejowski et al. postulieren, dass der Frakturspalt nach Einbringen des Transplantats in den Knochendefekt idealerweise weniger als 2 mm betragen sollte, um eine enchondrale Ossifikation zu induzieren [26].

Die knöcherne Defektauffüllung erfolgt, entsprechend der aktuellen Datenlage, zumeist durch Verwendung autologer Transplantate. Diese stammen mehrheitlich aus dem Beckenkamm oder werden mithilfe der RIA-Technik aus dem Femur oder der Tibia gewonnen. Einige kleinere Fallserien berichteten über die additive Zugabe **osteoinduktiver Wirkstoffe**

osteoinduktiver Wirkstoffe

wie beispielsweise von BMP‑2, BMP‑7, Knochenmarkaspiratkonzentrat („bone marrow aspirate concentrate“, BMAC) oder plättchenreichem Plasma (PRP). Xenogene oder synthetische Knochenersatzmaterialien fanden vergleichsweise wenig Erwähnung [18, 19, 20, 22, 27].

## Komplikationen

Trotz hoher Erfolgsraten der Knochenheilung im Rahmen der Anwendung der Masquelet-Technik zur Rekonstruktion knöcherner Defekte ist die Kenntnis der Gründe eines potenziellen Versagens der IMT essenziell. Drei Arten des Versagens lassen sich unterscheiden: septisch, mechanisch und biologisch [22].

Die **Infektpersistenz**

Infektpersistenz

einer aufgrund eines insuffizienten knöchernen Débridements, einer fehlgeschlagenen Weichteilrekonstruktion oder einer inadäquaten Antibiotikatherapie stellt die häufigste Ursache für das Versagen der IMT dar. Ein suffizientes Débridement ist sowohl in der ersten Phase (vor der Zement-Spacer-Interposition) als auch in der zweiten Phase, die eine weitere Gelegenheit zur Entfernung von avaskulärem und potenziell kontaminiertem Knochen darstellt, essenziell [17].

Eine unzureichende mechanische Stabilität im Rahmen der zweiten Phase stellt die zweite Ursache für ein Versagen der IMT dar. Ein Mangel an mechanischer Stabilität beeinträchtigt die Revaskularisierung und knöcherne Inkorporation des Transplantats, was zu einer aseptischen Pseudarthrose und einem anschließenden **Implantatversagen**

Implantatversagen

führen kann [14, 17]. Die Pseudarthrose tritt häufig zwischen dem Transplantat und dem distalen Knochenende auf [28]. Masquelet et al. postulierten daher, dass es von entscheidender Bedeutung sei, dass der Knochenzement die knöchernen Resektionsränder in der ersten Phase um 2 oder 3 cm umhüllt und eine abschließende Dekortikation der knöchernen Resektionsränder im Rahmen der zweiten Phase unter Beachtung der umgebenden induzierten Membran erfolgen müsse ([1, 20]; Abb. 1a aus dem Beitrag von Hückstädt et al. in der vorliegenden Ausgabe). Gleichwohl sollte eine zu rigide Fixation vermieden werden, um die sekundäre Kortikalisierung des Transplantats nicht zu kompromittieren. Die Autoren beschrieben zudem eine unzureichende Füllung der IM-Kavität als weitere Ursache für ein mechanisches Versagen. Insbesondere im Bereich der oberen Extremität besteht bei fehlender stabiler Verankerung des Transplantats das Risiko eines **Sedimentationseffektes**

Sedimentationseffektes;

dies gilt als Risikofaktor für das Ausbilden einer Pseudarthrose oder einer Refraktur im proximalen Teil des rekonstruierten Knochensegmentes [20].

Das biologische Versagen der IMT entspricht einer **ausbleibenden Revaskularisierung**

ausbleibenden Revaskularisierung

des Transplantats trotz erfolgreicher Infekteradikation und suffizienter mechanischer Stabilität. Diese Versagensform basiert laut Mathieu et al. auf einem inadäquaten Inhalt oder einem ungeeigneten inneren Milieu der IM [22].

### Merke

Einem Versagen der Masquelet-Technik liegt meist einer der folgenden Ursache zugrunde: septisch, mechanisch oder biologisch.

## Klinische Fallbeispiele

Die IMT erfreut sich 24 Jahre nach ihrer Erstbeschreibung zunehmender Popularität und unterliegt zahlreichen Modifikationen. In den Kliniken der Autoren wurden bis zum gegenwärtigen Zeitpunkt mehr als 300 Patienten mittels IMT behandelt. Die ursprüngliche Technik unterlag auch in eigener Hand mehreren Modifikationen.

**Abb. 3:**
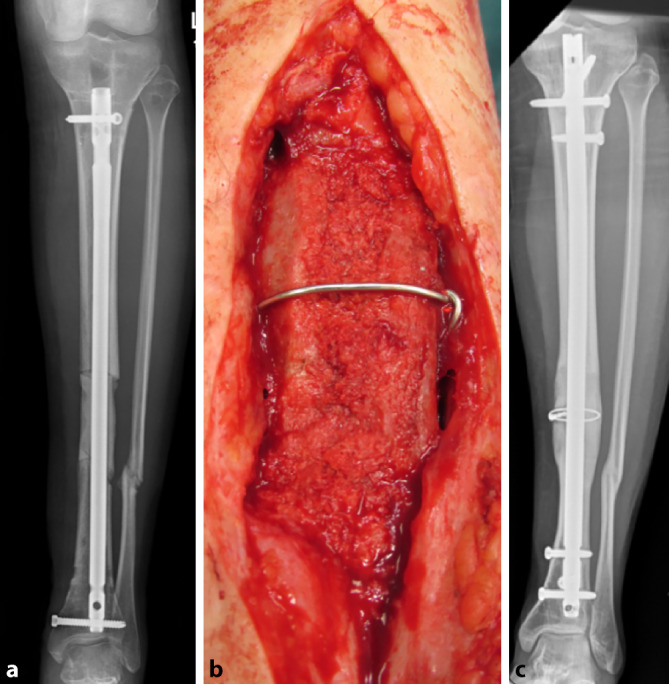
**a** 6 cm langer Segmentdefekt des Tibiaschaftes, temporäre Osteosynthese durch Marknagel (Segmentresektion bei implantatassoziierter Osteomyelitis). **b** Masquelet-Technik durch 2 bikortikale Beckenkammspäne (press fit), gesichert durch Cerclage und Beckenkammspongiosa. **c** Röntgenbild nach 2,5 Jahren, vollständige Konsolidierung und Remodeling

Kleinere knöcherne Defekte (untere Extremität: bis etwa 5 cm, obere Extremität bis etwa 10 cm) werden im eigenen Patientenkollektiv durch eine Kombination aus autologen bi- oder trikortikalen Beckenkammspänen und interner Osteosynthese rekonstruiert (Abb. 3a–c).

Das **Entnahmevolumen**

**Abb. 4:**
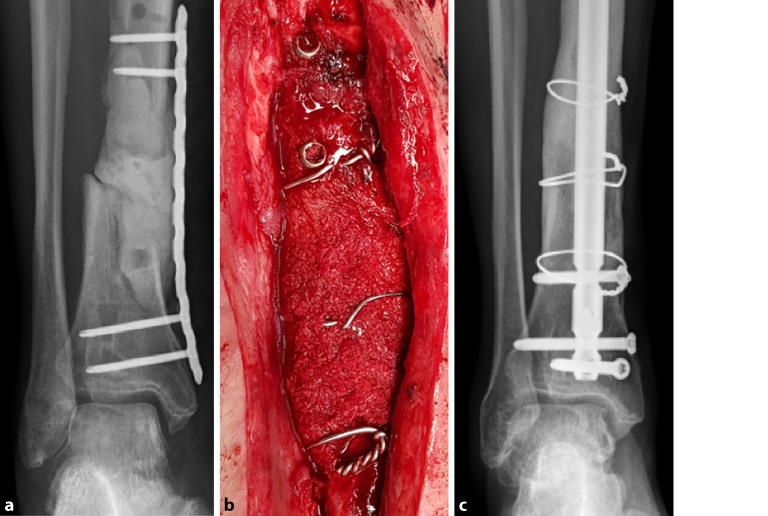
**a** Segmentdefekt der distalen Tibia (3 cm ventral, 9 cm dorsal). **b** Masquelet-Technik durch einen trikortikalen Beckenkammspan (ventral) und einen halbierten angepassten dekortizierten thermodesinfizierten Spenderfemurkopf, ummantelt mit autologer Spongiosa vom Beckenkamm, Sicherung durch Cerclagen. **c** Konsolidierung und Remodeling nach 1,5 Jahren

Entnahmevolumen

des Beckenkammspongiosa stellt den limitierenden Faktor dar, sodass größere Knochendefekte nicht mit dieser Variante behandelbar sind. Reicht das Volumen zur Deckung des knöchernen Defektes nach Verwendung bi- oder trikortikaler Beckenkammspäne allein nicht aus, können additiv spongiöse Blöcke aus **thermodesinfizierten Spenderfemurköpfchen**

thermodesinfizierten Spenderfemurköpfchen,

die zuvor mit autologer Spongiosa des Beckenkammes ummantelt werden, interponiert werden (Abb. 4a–c).

Die Verwendung des RIA-Systems ermöglicht die Entnahme größerer Mengen Spongiosa [29, 30, 31]. Knöcherne Defekte bis zu einer Größe über 20 cm werden im eigenen Patientenkollektiv durch die Kombination aus autologer (RIA-)Spongiosa und soliden angepassten thermodesinfizierten Femurköpfen in Verbindung mit stabilen internen Osteosynthesen behandelt. Die Entnahme der RIA-Spongiosa erfolgt entweder ortho- oder retrograd aus dem ipsi- oder kontralateralen Femur, alternativ aus der Tibia. Die Menge der entnommenen Spongiosa richtet sich nach der Größe des knöchernen Defekts. Eine Defektstrecke von 4 cm (entspricht einem Femurkopf) bedarf beispielsweise einer Menge von 10 cm^3^ Spongiosa. Für einen 20 cm Defekt werden also etwa 50 ml RIA-Spongiosa benötigt.

Die Anzahl der verwendeten allogenen Femurköpfe orientiert sich ebenfalls an der Größe des knöchernen Defekts. Nach Entknorpeln und Dekortizieren der allogenen Transplantate entstehen spongiöse Zylinder einer Länge zwischen 3 und 6 cm. Für einen Segmentdefekt von 20 cm werden 5 Femurköpfe benötigt. Erfolgt eine interne Fixation mithilfe der **Marknagel-Osteosynthese**

**Abb. 5:**
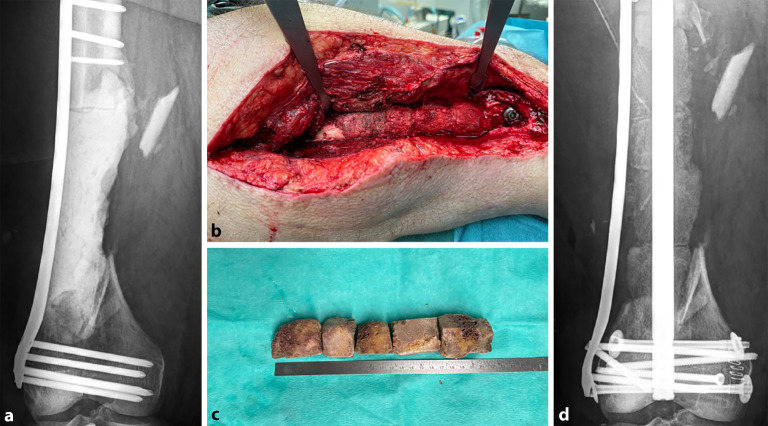
**a** Ein 18 cm Segmentdefekt des distalen Femurs, temporäre winkelstabile Platte. **b,** **c** Masquelet-Technik durch 5 kanülierte dekortizierte thermodesinfizierte Femurköpfe in Kombination mit RIA-Spongiosa (40 ml). **d** Postoperatives Röntgenbild mit hochstabiler Osteosynthese durch Marknagel und additive winkelstabile Platte

Marknagel-Osteosynthese,

werden die Femurköpfe kanüliert und 1 mm über den Durchmesser des Marknagels aufgebohrt. Die spongiösen Zylinder werden mit der gewonnenen RIA-Spongiosa ummantelt. Der Marknagel wird bis zur Zone des Segmentdefektes eingebracht, und die Femurköpfe werden auf den Nagel aufgefädelt. Dabei ist auf eine ausreichende Auffüllung der Kontaktflächen zwischen dem Knochen und dem Femurkopf sowie zwischen den Femurköpfen untereinander mithilfe von RIA-Spongiosa zu achten [32, 33]. Bei ausreichend vorhandener RIA-Spongiosa kann diese zur Deckung kleinerer Defekte bzw. an die Kontaktzonen der Masquelet-Plastik angelagert werden. Sofern möglich, kann Kompression auf Osteosynthese und Masquelet-Plastik aufgebracht werden (Abb. 5a–d).

Aus Erfahrung der Autoren ist die absolute Stabilität von Osteosynthese und Masquelet-Plastik essenziell, um eine knöcherne Fusion zu erreichen. Somit sollten möglichst solide auto- oder allogene Knochentransplantate Anwendung finden, die, wann immer möglich, in **Press-fit-Technik**

Press-fit-Technik

eingebracht werden, um in Kombination mit dem Osteosyntheseimplantat eine möglichst hohe Primärstabilität zu erreichen.

Bei Bedarf können diesbezüglich neben intramedullären Marknägeln additive winkelstabile Platten verwendet werden. In gelenknahen Bereichen erfolgt der Einsatz **winkelstabiler Doppelplattenosteosynthesen**

winkelstabiler Doppelplattenosteosynthesen.

Unter Anwendung der oben genannten Verfahren und Prinzipien konnten im eigenen Patientenkollektiv mit der MT knöcherne Defekte von Defektdistanzen zwischen 2,5 und 22 cm erfolgreich rekonstruiert werden. Die primäre Konsolidierungsrate beträgt über 90 % und ist mit den Ergebnissen der aktuellen Literatur vergleichbar.

## Fazit für die Praxis

- Die Masquelet-Technik ist ein zweizeitiges Verfahren zur Rekonstruktion großer knöcherner Defekte verschiedener Ätiologien.
- Der Erfolg dieses Verfahrens ist von einer suffizienten Analyse der vorliegenden Situation und einer geeigneten chirurgischen Taktik abhängig.
- Die Grundprinzipien des Diamond-Konzeptes sind zwingend zu beachten.
- In Verbindung mit einer stabilen internen Osteosynthese stellt die Kombination solider Spenderfemurköpfe mit Spongiosa, die mithilfe der Reamer-Irrigator-Aspirator(RIA)-Technik gewonnen wurde, eine vielversprechende Modifikation der Masquelet-Technik dar.
- Die dadurch gewährleistete primäre Übungsstabilität und Teilbelastung führen frühzeitig zur knöchernen Konsolidierung und Vollbelastung.
- Die Kenntnis der Gründe für ein Versagen der Masquelet-Technik ist essenziell. Folgende Ursachen lassen sich unterscheiden: septisch, mechanisch und biologisch.

## CME-Fragebogen

Welches Verfahren kommt bei der Rekonstruktion segmentaler Knochendefekte zur Anwendung?

Kortikalisdistraktion

Extrakorporale Stoßwellentherapie

Reamer Irrigator Aspirator (RIA) aus dem Markraum langer Röhrenknochen

Steigbügelplastik

Zugabe von Lipoproteinen

In der Notaufnahme stellt sich Ihnen ein Patient mit einer implantatassoziierten Osteomyelitis vor. Sie leiten die notwendigen Schritte ein, entfernen im ersten operativen Eingriff das einliegende Osteosynthesematerial und resezieren das infizierte Knochensegment. Im Rahmen dieses Ersteingriffs erfolgt die temporäre Interposition eines Polymethylmethacrylat(PMMA)-Spacers in den knöchernen Defekt in Vorbereitung auf die Masquelet-Technik. Was erwarten Sie dadurch?

Die Masquelet-Technik ist ein einzeitiges Verfahren zur Rekonstruktion segmentaler Knochendefekte

Die Möglichkeit des Verzichts auf eine zusätzliche Osteosynthese

Um den PMMA-Spacer wird direkt die Bildung neuen Knochens induziert

Die Bildung einer gut vaskularisierten Pseudosynovialmembran

Eine sofortige Vollbelastung der verletzten Extremität

Welches ist ein Kriterium des Diamond-Konzeptes im Rahmen der Behandlung von Knochendefekten und Pseudarthrosen?

Osteokonduktion

Osteointeraktion

Osteotransduktion

Osteokonvektion

Osteotransfixation

Welche Signalmoleküle stellen essenzielle Bestandteile der induzierten Membran bei der Masquelet-Technik dar?

Metalloproteasen

Alpha-1-Fetoprotein

Wachstumsfaktoren

Lipoproteine

Tau-Protein

Welches Verfahren wird in der originären Masquelet-Technik beschrieben?

Reamer-Irrigator-Aspirator(RIA)-Technik

Autologe Spongiosaentnahme vom Beckenkamm

Kallusdistraktion über Segmenttransport

Zugabe von Knochenwachstumsfaktoren

Bioaktive Gläser und Keramik auf Kalziumbasis

Was stellt ein typisches Problem der solitären Verwendung von Reamer-Irrigator-Aspirator(RIA)-Spongiosa im Rahmen der Masquelet-Technik dar?

Die RIA-Spongiosa enthält zu wenige osteogene Zellen.

Die RIA-Spongiosa enthält zu wenige Knochenwachstumsfaktoren.

Die RIA-Spongiosa führt typischerweise zu einer Hyperostose.

Die RIA-Spongiosa kann bei solitärer Verwendung zu einem Sedimentationseffekt führen.

Die RIA-Spongiosa stellt das optimale Nährmedium für Bakterien dar.

Sie planen die Anwendung der Masquelet-Technik im Rahmen einer implantatassoziierten Osteomyelitis nach Nagelung einer Tibiafraktur. Was stellt den ersten Schritt dar?

Nach Gewinnung einer perkutanen Probenentnahme zunächst Durchführen einer 6‑wöchigen i.v.-Antibiotikabehandlung gemäß Antibiogramm.

Die komplette Entfernung des Osteosynthesematerials, ein Weichteil-Débridement und Resektion von minderperfundiertem Knochen.

Zunächst nur die komplette Metallentfernung, um einen konservativen Therapieversuch zu unternehmen.

Im Rahmen des einzeitigen Verfahrens erfolgt die sofortige Defektauffüllung mit autologer Beckenkammspongiosa.

Der Nagel verbleibt in situ, und es wird ein Débridement im Bereich des infizierten Knochens durchgeführt.

Welche Körperregion ist eine typische Entnahmelokalisation autologer Spongiosa?

Vorderer Beckenring

Radius

Ulna

Sternum

Calcaneus

Sie behandeln einen Patienten mit einem knöchernen Segmentdefekt. Im Rahmen der präoperativen Aufklärung erläutern Sie die verschiedenen Behandlungsoptionen: Segmenttransport bzw. Masquelet-Technik (MT). Worin liegen die wesentlichen Unterschiede der beiden Verfahren?

Die Konsolidierungsrate im Rahmen des Segmenttransportes ist höher als die der MT.

Die Technik des Segmenttransportes ist einfacher als die der MT.

Der Segmenttransport kann nur an Femur und Tibia durchgeführt werden.

Die Behandlungsdauer der MT ist kürzer als die des Segmenttransportes.

Der Segmenttransport kann nur über einen Fixateur externe erfolgen.

Worin liegt die Modifikation der ursprünglichen Masquelet-Technik?

In der überbrückenden Stabilisierung mittels Fixateur externe.

Es wird eine sofortige Vollbelastung durchgeführt, um die Knochenneubildung anzuregen.

Interne Osteosynthesen kommen im Ersteingriff zunehmend zur Anwendung.

In der ersten Phase erfolgen ein Weichteil-Débridement und Resektion von minderperfundierten Knochenarealen.

In der zweiten Phase wird die Defektauffüllung durchgeführt.
